# Silicon Promotes Agronomic Performance in *Brassica napus* Cultivated under Field Conditions with Two Nitrogen Fertilizer Inputs

**DOI:** 10.3390/plants8050137

**Published:** 2019-05-22

**Authors:** Philippe Laîné, Cylia Haddad, Mustapha Arkoun, Jean-Claude Yvin, Philippe Etienne

**Affiliations:** 1 Normandie Université, UniCaen, INRA, UMR 950 EVA, SFR Normandie Végétal (FED4277), 14000 Caen, France; philippe.laine@unicaen.fr (P.L.); cylia.haddad@yahoo.fr (C.H.); 2 Centre Mondial de l’Innovation, Groupe Roullier, 35000 Saint Malo, France; mustapha.arkoun@roullier.com (M.A.); JeanClaude.Yvin@roullier.com (J.-C.Y.)

**Keywords:** agronomic indexes, micronutrients, rapeseed, silicon, yield

## Abstract

To limit the environmental pollution associated with intensive nitrogen (N) fertilizer usage, alternative cultural practices must be considered for crops requiring high N inputs such as rapeseed. In this context, the effects of silicon (Si) supply on the agronomic performance of rapeseed cultivated under field conditions with two N fertilizer levels (60 and 160 kg ha^−1^) were studied. Results showed that Si supplied in the form of silicic acid (12 kg ha^−1^) has no effect on the agronomic performance of plants cultivated with the lower N input. In contrast, in plants fertilized with 160 kg N ha^−1^, Si supply promotes the preservation of green leaves (until the flowering stage) and at harvest stage, increases biomass, yield, and seed micronutrient concentrations (especially cobalt and iron). The agronomic indexes show that the increase in seed yield is related to a better uptake of N from the soil by Si-treated plants, but is not an improvement in N mobilization towards the seeds. This study showed that Si supply combined with high N inputs (160 kg ha^−1^) improves usage of N fertilizer and yield. The possibility that a Si supply could allow for a reduction in N input without altering the yield of rapeseed is discussed.

## 1. Introduction

Silicon (Si) is the second most abundant element in the soil after oxygen and comprises about 28% of the Earth’s crust [1,2]. Si is mainly present in soil as crystalline aluminosilicates, an insoluble form not directly available to plants [3]. In contrast, silicic acid (Si(OH)_4_), the soluble form of silicon available for plants is very scarce in most soils (with concentrations in soil solution ranging from 0.1 to 0.6 mM) [4]. Silicic acid is taken up by plants via aquaporins belonging to the Nodulin-26-like intrinsic proteins 2 (NIP2) group with a G-S-G-R SF pore (NIP-III aquaporins) known as the Si influx transporter (Lsi1) [5,6].Vascular plant species have different abilities to take up and accumulate Si, leading to classification into three groups according to the Si concentration: “strong accumulators”, such as rice, with a Si content reaching 15% of dry weight (DW), “intermediate accumulators”, such as rye, oats, or wheat, with a Si content comprising 1%–3% of DW, and finally “weak Si accumulators” like rapeseed with a Si content of less than 0.1% of DW [7,8,9,10]. In Brassicaceae species, this low Si accumulation is explained by the lack of Si transporters (especially NIP-IIIs), as recently demonstrated by Sonah al. [6]. Although Si is not considered as an essential element for higher plants, several beneficial effects have been shown, especially for the alleviation of various plant biotic and abiotic stresses [11]. For example, Si application reduces the severity of fungal diseases such as powdery mildew in barley [12] and other diseases, such as blast and brown spot in rice [13,14]. Moreover, some studies have shown that Si supply improves plant tolerance to toxic metals such as cadmium, aluminum, and chrome [15,16] and salt stress [17]. Furthermore, Haddad et al. [10] indicated a beneficial effect of Si treatment in alleviating damage associated with nitrogen (N) deficiency in rapeseed. These authors showed that Si supply modulates the root expression of a large panel of genes [18], promotes a stronger N uptake associated with induction of root nitrate transporters, and delays leaf senescence in plants cultivated under N deficiency [10]. This increase in the leaf life span was particularly interesting because Malagoli et al. [19] reported that an increase in the leaf life span of rapeseed could allow for better synchronization between leaf N remobilization and seed N filling, and could lead to a greater seed yield. Taking into account these effects, but also the low availability of silicic acid in most soils, a Si supplementation with Si-based fertilizers could be particularly beneficial for promoting growth and alleviating stress in agronomic crops.

The aim of this study was to investigate the agronomic performance of rapeseed plants cultivated under field conditions and fertilized with two N inputs (60 and 160 kg ha^−1^) and supplied or not with a form of Si available to plants (12 kg Si ha^−1^ in the silicic acid form). For this, the effects of Si have been monitored in the whole plant biomass and during leaf senescence at the flowering stage (G1 stage, [20]), but also in yield components, seed quality, and N use efficiency indexes such as the nitrogen harvest index (NHI), agronomic efficiency (AE), and agronomic nitrogen recovery (ANR) at the final harvest stage (G5 stage). 

## 2. Materials and Methods

### 2.1. Site, Climatic Conditions, and Lysimeter Description

The field experiments were conducted in a lysimeter system located at Lieury (France, Calvados, 00° 00’ 34.3” W, 48° 59’ 24.2” N) from 17 September, 2016 to 7 July, 2017. The lysimeter device comprised of 15 polyester resin lysimeter boxes with an area of 1.96 m² (1.4 m × 1.4 m), and a depth of 1.10 m. Rainfall and air temperature were measured continuously at the meteorological station at the Lycée Le Robillard in Lieury (France, Calvados; supplemental data S1). The mean minimum and maximum air temperatures were 5.86 and 16.23 °C, respectively, and the total precipitation was 508.20 mm during the period of winter oilseed rape cultivation (2016–2017).

Each lysimeter box was filled with an arable Calcaric Cambisol soil with 4.3% organic matter in the top 0–20 cm, basic pH, and calcium carbonate content that increased with depth (Table 1).

### 2.2. Experimental Design

**Figure 1:**
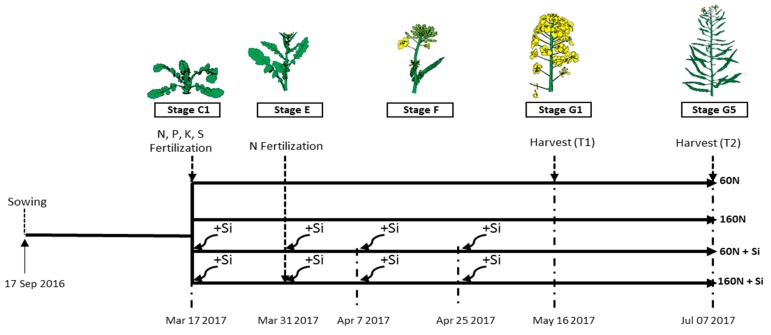
Experimental design used to study the effect of silicon (Si) on the agronomic performance of *Brassica napus* L. cultivated into lysimeter boxes. When plants were at stage C1 (March 17, 2017), fertilization combining nitrogen (N: 30 or 80 kg ha^−1^ for 60 and 160 N treatments, respectively), phosphorus (P: 50 kg ha^−1^), potassium (K: 50 kg ha^−1^) and sulfur (S: 30 kg ha^−1^) was applied. At stage E (March 31 2017), a second N fertilization with 30 or 80 kg N ha^−1^ was applied to the 60 and 160 N treatments, respectively. In addition, for each N treatment (60 and 160 N), fertilization with Si (Si: 12 kg ha^−1^ as Si(OH)_4_) was applied in four times (March 17 and 31 and April 7 and 25 2017: 60 N + Si and 160 N + Si) or not (controls: 60 N and 160 N). For all treatments, plants were harvested at T1 and T2 corresponding to the G1 and G5 stages, respectively.

Seeds of rapeseed (*Brassica napus* L. var. ‘Citizzen’) were sown (7 September, 2016) as previously described by Génard et al. [21]. At stage B4 (after the appearance of the first 4 leaves), young plants were carefully transplanted into the lysimeters (2 m^2^ of area), with a density of 60 plants per lysimeter. At stage C1 (rosette stage, which was March 17, 2017), the 15 lysimeter boxes were randomly separated into five batches and each batch was composed of three lysimeter boxes. As usually recommended [22], all lysimeters were fertilized with: 40 kg P ha^−1^ (as P_2_O_5_), 50 kg K ha^−1^ (as K_2_O), and 30 kg S ha^–1^ (as MgSO_4_). Among these 15 lysimeter boxes, three of them did not receive N fertilization (control: 0 kg N ha^−1^; Supplementary Figure S1). Among the twelve remaining lysimeter boxes, six received 60 kg N ha^−1^ (60 N) and the others received 160 kg N ha^−1^ (160 N). Nitrogen was supplied as ammonium nitrate fractioned into two N inputs (twice 30 or 80 kg N ha^−1^ on 17 March, 2017 (C1 stage) and March 31, 2017 (stage E) for plants fertilized with 60 and 160 kg N ha^−1^ (Figure 1). Finally, half of the lysimeters boxes fertilized with either 60 or 160 kg N ha^−1^ were supplied with 12 kg Si ha^−1^ (60 N + Si and 160 N + Si) in the form of Si(OH)_4_, which was fractioned into four inputs: 1 kg ha^−1^ at stage C1 (17 March, 2017), 1 kg ha^−1^ at stage E (bolting stage, March 31, 2017), and two inputs of 5 kg ha^−1^ during stage F (flowering stage, April 7 and 25, 2017). In contrast, the remaining 60 N and 160 N lysimeters were considered as controls of Si supply (60 N and 160 N) and did not receive Si inputs (Figure 1). The dose of Si was determined as the amount required to obtain a Si concentration of at least ≥0.1% of dry weight tissues (concentration usually found in rapeseed) if the Si supplied is totally uptaken by plants. The silicic acid solution (Si(OH)_4_ was prepared by passing sodium silicate through cation exchange resin (Amberlite IR-120 hydrogen form, Sigma-Aldrich, Saint-Quentin-Fallavier, France) and its concentration was determined using the colorimetric method previously described by Haddad et al. [10]. For the first two fertilizations with Si, a 12 L Si(OH)_4_ solution at 57 mg L^−1^ was applied, and for the last two fertilizations with Si, a 20 L of Si(OH)_4_ solution (pH 6) at 171.5 mg L^−1^ was applied uniformly to each set of lysimeter boxes with a watering can with sprinkler system. At each date of Si supply, the same volumes (12 or 20 L) of water were applied to the lysimeter boxes without Si. 

For all treatments, plants cultivated on half of the respective lysimeter area (i.e., 30 plants corresponding to 1 m^2^) were harvested at the G1 stage (rosette stage; harvest T1 on May 16, 2017) and the G5 stage (ripening stage; harvest T2 on July 7, 2017). At each harvest date, plant organs (taproots, stems, leaves and fallen leaves, and pods (only at T2)) were separated, weighed, and oven dried for 48 h at 60 °C for DW determination and then ground to fine powder for further chemical analysis. 

### 2.3. Determination of the Percentages of Green and Senescent Leaves

At harvest T1, all leaves of the whole plants were collected and total leaf dry weight was determined. Then the leaves were batched into fully green leaves and senescent leaves characterized by the presence of visual yellow areas. The dry weight of each batch was determined and the percentage of green or senescent leaves on plants was calculated.

### 2.4. Elemental Analysis

For N analysis, aliquots of each dried sample (pods, seeds, taproots, stems, and leaves of plants at T2) were placed into tin capsules using a microbalance and the total N concentration was determined with a continuous flow isotope ratio mass spectrometer (IRMS, Horizon, NU Instruments, Wrexham, United Kingdom) linked to a C/N/S analyzer (EA3000, Euro Vector, Milan, Italy). 

In mature seeds, macronutrients (N, P, K, S, Ca, Mg) and microelements (Zn, Mn, B, Cu, Mo, Se, Ni, Fe, Co) were quantified by high-resolution inductively coupled plasma mass spectrometry (HR ICP-MS, Thermo Scientific, Element 2^TM^). Dry matter of each of the samples (40 mg) was suspended with 800 µL of concentrated HNO_3_, 200 µL of H_2_O_2_, and 1 mL of Milli-Q water. All samples were then spiked with three internal standard solutions containing gallium, rhodium, and iridium with final concentrations of 5, 1, and 1 µg L^−1^, respectively. After microwave acidic digestion (Multiwave ECO, Anton Paar, les Ulis, France), all samples were diluted with 50 mL of Milli-Q water to obtain solutions containing 2.0% (*v*/*v*) nitric acid. Before HR ICP-MS analysis, samples were filtered at 0.45 µm using a Teflon filtration system (Digifilter, SCP Science, Courtaboeuf, France). Quantification of each element was performed using external standard calibration curves and concentrations were expressed in mg g^−1^ and µg g^−1^ of seed DW for macronutrients and microelements, respectively.

### 2.5. Determination of Oil, Protein, and Glucosinolate Concentrations in Mature Seeds with Near Infrared Spectroscopy (NIRS)

Near infrared spectroscopy analysis was performed as previously described by Génard et al. [21]. Briefly, intact seeds (5 g) were placed in a standard ring cup and were scanned on a near infrared monochromator spectroscopy system equipped with a transport module in the reflectance mode (model 65000, FOSS NIR System Inc., Silver Spring, MD, USA). Results were obtained from an external calibration established for oil, protein, and total glucosinolate (GLS) concentrations (CRAW, Gembloux, Belgium). Oil and protein concentrations were expressed in % of seed dry matter (DW) and glucosinolate concentrations were expressed in µg g^−1^ of seed DW. 

### 2.6. Determination of Nitrogen Use Efficiency Indexes

Agronomic efficiency (AE), the nitrogen harvest index (NHI), and agronomic nitrogen recovery (ANR) were calculated to characterize the nitrogen use efficiency of *Brassica napus* fertilized with two levels of N inputs and supplied or not with Si. 

Agronomic efficiency (AE), which represents the efficiency of the crop to convert the applied nitrogen to seed yield, was calculated as follows:

$$\mathrm{AE}\;=\;\frac{[\left(\mathrm{SDW}\;\mathrm{with}\;\mathrm{fertilizer}\;\left(60\;N,60\;N\;+\mathrm{Si},\;160\;N\;\mathrm{or}\;160\;N\;+\;\mathrm{Si}\right)\;-\;\mathrm{SDW}\;\mathrm{without}\;N\;\mathrm{fertilizer}\;\left(0\;N\right)\right]}{\mathrm{Nf}\;\left(60\;\mathrm{or}\;160\;N\right)}\times 100$$

where SDW and Nf correspond to seed dry weight and the nitrogen supply from fertilizer, respectively. The value of SDW without N fertilizer (0 N) is presented in supplemental data S2.

ANR, defined as the efficiency of nitrogen capture from soil was calculated as follows:

$$\mathrm{ANR}\;=\;\frac{[\left(\mathrm{Nt}\;\mathrm{with}\;\mathrm{fertilizer}\;\left(60\;N,\;60\;N\;+\;\mathrm{Si},\;160\;N\;\mathrm{or}\;160\;N\;+\;\mathrm{Si}\right)\;-\;\mathrm{Nt}\;\mathrm{without}\;N\;\mathrm{fertilizer}\;\left(0\;N\right)\right]}{\mathrm{Nf}\;\left(60\;\mathrm{or}\;160\;N\right)}\times 100$$

where Nt and Nf correspond to the total N amount in the whole plant and the N amount supplied from fertilizer, respectively. The vegetative and seed N amounts from plants cultivated without N fertilizer (supplemental data S2) have been added to calculate Nt.

NHI, which represents the efficiency of nitrogen mobilization towards the seeds, was calculated using the following equation:

$$\mathrm{NHI}\;=\;\frac{\left[\mathrm{Ns}\;\left(60\;N,\;60\;N\;+\;\mathrm{Si},\;160\;N\;\mathrm{or}\;160\;N\;+\;\mathrm{Si}\right)\right]}{\left[\mathrm{Nt}\;\left(60\;N,\;60\;N\;+\;\mathrm{Si},\;160\;N\;\mathrm{or}\;160\;N\;+\;\mathrm{Si}\right)\right]}$$

where Ns and Nt correspond to total N in seed and in whole plants fertilized with 60 and 160 kg N ha^−1^ and supplied (60 N + Si or 160 N + Si) or not (60 N or 160 N) with Si (12 kg ha^−1^), respectively. 

### 2.7. Statistical Analysis

The experiment was performed with three replicates for each crop (0 N, 60 N, 160 N, 60 N + Si, and 160 N + Si). The resulting variations in data are expressed as the mean ± standard error (SE). Data were analyzed using analysis of variance (ANOVA), after verifying compliance of normality, and significantly different means between treatments were separated with the Tukey’s multiple range test (*p* ≤ 0.05).

## 3. Results

### 3.1. Effect of Silicon Supply on Plant Growth and Leaf Development

**Figure 2:**
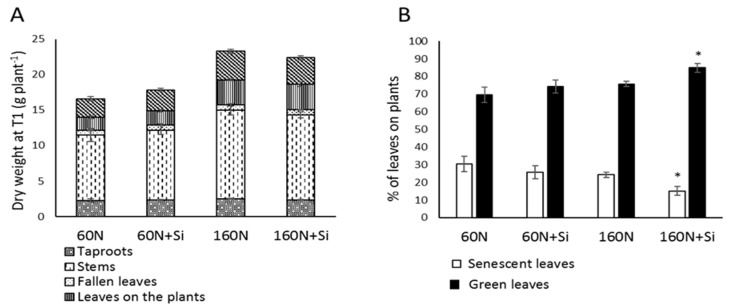
Compartment and total biomasses (**A**) and percentage of green and senescent leaves (**B**) of rapeseed plants harvested at T1 (G1 stage). Plants were grown in lysimeters with 60 (60 N) or 160 kg of N ha^−1^ (160 N) and supplied with or without Si (12 kg ha^−1^: 60 N + Si and 160 N + Si). Values correspond to the mean ± SE for *n* = 3. The data obtained from plants treated with silicon (60 N + Si and 160 N + Si) were compared to their respective controls. * indicates a significant difference at *p* ≤ 0.05.

For a given level of nitrogen fertilization (60 or 160 kg N ha^−1^) at the G1 developmental stage (i.e., flowering, harvest T1) in *Brassica napus*, Si supply (12 kg Si ha^−1^) had no significant effect on the biomass of different compartments or on the total biomass of plants. Thus, the cumulative biomasses of plants were 18.91.0 ± 1.74, 18.47 ± 0.95, 24.18 ± 1.24 and 25.46.0 ± 1.33 g for 60 N, 60 N + Si, 160 N and 160 N + Si plants, respectively (Figure 2A). Moreover, for plants cultivated with 60 kg N ha^−1^, Si supply had no significant effect on the distribution of green (69.5 and 74.2% for 60 N and 60 N + Si, respectively) and senescent leaves (30.5 and 25.8% 60 N and 60 N + Si, respectively). In contrast, for plants cultivated with 160 kg N ha^−1^, the distribution of green and senescent leaves was significantly different in plants treated with Si (160 N + Si) compared to the control (160 N). Thus, in plants cultivated with 160 N, Si supply increased the percentage of green leaves (84.9% vs. 75.7% in 160 N + Si and 160 N plants, respectively) and decreased the percentage of senescent leaves (15.1% vs. 24.3% in 160 N + Si and 160 N plants, respectively) (Figure 2B).

**Figure 3:**
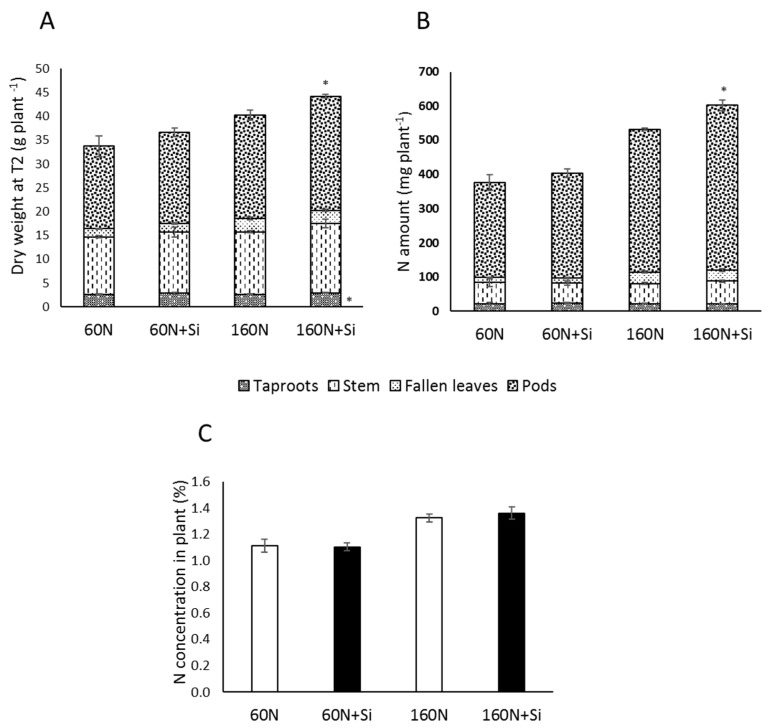
Compartment and total biomasses (**A**), total nitrogen amount (**B**) and N concentration (**C**) in rapeseed plants harvested at T2 (G5 stage). Plants were grown in lysimeter with 60 (60 N) or 160 kg of N ha^−1^ (160 N) and supplied with or without Si (12 kg Si ha^−1^: 60 N + Si and 160 N + Si). Values correspond to the mean ± SE for *n* = 3. The data obtained from plants treated with silicon (60 N + Si and 160 N + Si) were compared to their respective control. * indicates a significant difference at *p* ≤ 0.05.

**Figure 4:**
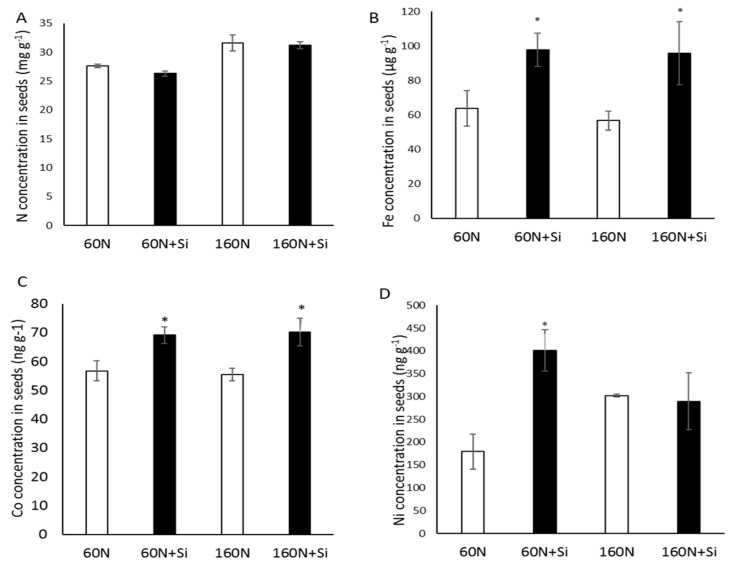
Nitrogen (**A**), Iron (**B**), Cobalt (**C**) and Nickel (**D**) concentrations in mature seeds of plants harvested at T2 (G5 stage). Plants were grown in lysimeters with 60 (60 N) or 160 kg of N ha^−1^ (160 N) and supplied with or without Si (12 kg ha^−1^: 60 N + Si and 160 N + Si). Values correspond to the mean ± SE for *n* = 3. The data obtained from plants treated with silicon (60 N + Si and 160 N + Si) were compared to their respective controls. * indicates a significant difference at *p* ≤ 0.05.

At the G5 developmental stage (corresponding to mature seeds, harvest T2), Si supply had no significant effect on the biomass of different compartments or on the total biomass of plants cultivated with 60 N. In plants cultivated with 160 N, Si supply led to an increase in taproot biomass (2.94 ± 0.03 vs. 2.67 ± 0.09 g plant^−1^ in 160 N + Si and 160 N plants, respectively) and whole plant biomass (45.13 ± 0.73 vs. 40.23 ± 1.33 g plant^−1^ in 160 N + Si and 160 N plants, respectively) (Figure 3A). Moreover, the Si supply had only a significant effect on plants fertilized with 160 kg N ha^−1^ (Figure 4B). However, it should be noted that the increase in the N amount in 160N + Si plants was not linked to an increase in their N concentration (Figure 3C) but rather to an increase in their total biomass (Figure 3B). 

### 3.2. Effect of Silicon Supply on Yield and Seed Quality Components

Si supply had no effect on the yield of plants fertilized with 60 kg N ha^−1^ (60 N), whereas it led to a significant increase in yield in plants cultivated with 160 kg N ha^−1^ (160 N). Thus, the yield of 160 N + Si plants reached 4247.20 ± 75.23 vs. only 3644.37 ± 182.48 kg ha^−1^ in the 160 N plants. Since the thousand-seed weight is similar between the Si treatments (Table 2), it is possible that the increase in seed biomass in 160 N + Si plants was due to a higher number of pods per plant and/or a higher number of seeds per pod. Moreover, Si supply had no significant effect on the quality components (such as thousand seed weight, glucosinolate, oil, and protein concentrations) of seeds from plants cultivated with 60 and 160 kg N ha^−1^ (Table 2).

### 3.3. Effect of Silicon Supply on Elemental Seed Composition

Using ICPMS, the concentrations of six macroelements (N, P, K, S, Ca, Mg) and nine microelements (Zn, Mn, B, Cu, Mo, Se, Ni, Fe, Co) were analyzed in seeds from plants cultivated with 60 or 160 N and supplied with or without Si. For a given N fertilization, Si supply had no effect on either the N concentration (Figure 4A) or on the concentrations of other macroelements (P, K, S, Ca, Mg; Supplementary Table S1) in seeds. Moreover, whatever the N fertilization used (60 or 160 kg ha^−1^), the Co and Fe concentrations in seeds of plants supplied with Si (60N + Si and 160N + Si) increased compared to their respective controls (60N and 160N) (Figure 4B,C). Therefore, Si supply increased the Ni concentration by 2.23-fold in seeds of plants grown with 60 kg N ha^−1^ (Figure 4D). For the other seven microelements, Si supply did not modify their seed concentrations under any of the N fertilization inputs (Supplementary Table S1). 

### 3.4. Effect of Silicon on the Nitrogen Use Efficiency Component of Plants 

**Figure 5:**
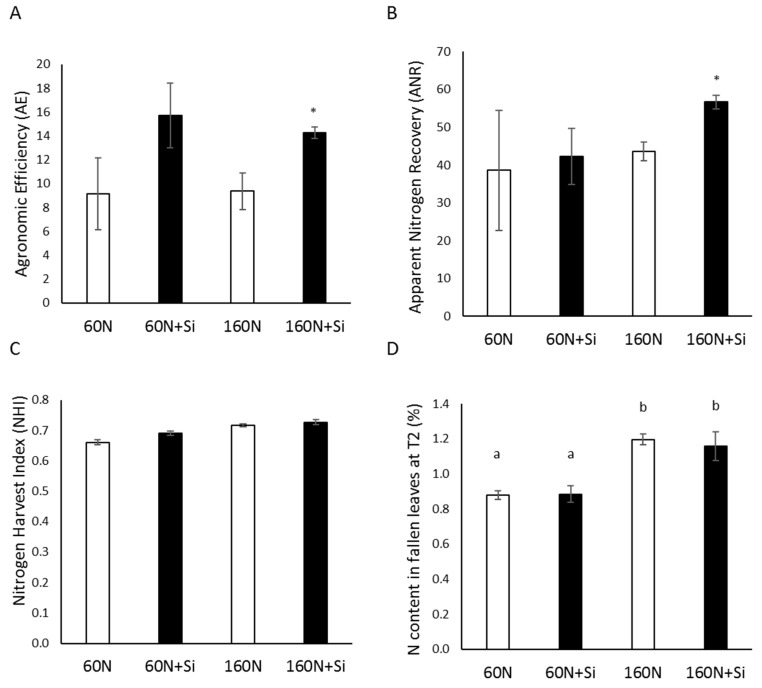
Agronomic efficiency of N fertilizer (**A**), apparent nitrogen recovery (**B**), the nitrogen harvest index (**C**) and N content in fallen leaves (**D**) of plants harvested at T2 (G5 stage). Plants were cultivated in lysimeters with 60 (60 N) or 160 kg of N ha^−1^ (160 N) and supplied with or without Si (12 kg of SiO_4_ ha^−1^; 60 N + Si, 160 N + Si). Values correspond to the mean ± SE for *n* = 3. The data obtained from plants treated with silicon (60 N + Si and 160 N + Si) were compared to their respective controls (60 N and 160 N). * indicates a significant difference at *p* ≤ 0.05 and different letters indicate significant difference between treatments at *p* ≤ 0.05.

For plants cultivated with 60 kg N ha^−1^ (60 N), Si supply had no significant effect on agronomic efficiency of N fertilizer (AE; Figure 5A), apparent nitrogen recovery (ANR; Figure 5B), nitrogen harvest index (NHI; Figure 5C), or residual N concentration in fallen leaves (Figure 5D). In contrast, when plants were cultivated with 160 kg N ha^−1^ (160 N), Si supply led to significant increases in AE and ANR of 1.5- and 1.3-fold, respectively. In addition, neither the NHI nor the residual N concentrations in fallen leaves were modified by the Si supply. Moreover, it was notable that the residual N concentrations in fallen leaves of plants cultivated with 60 kg N ha^−1^ (60 N and 60 N + Si) was significantly lower than those in fallen leaves from plants cultivated with 160 kg N ha^−1^ (160 N and 160 N + Si) and reached about 0.9% and 1.2%, respectively (Figure 5D).

## 4. Discussion

The developmental stages studied (flowering and final stages) showed that Si supply (12 kg Si ha^−1^) with the lower nitrogen input (60 kg N ha^−1^) had no significant effect on the growth, yield, or seed quality of rapeseed cultivated under field conditions (Figure 2 and Figure 3A; Table 2). This seems surprising in the light of a recent study performed by Haddad et al. [10] that demonstrated the beneficial effect of Si supply on young N-deprived rapeseeds over a short period (twelve days) under hydroponic conditions. This difference in the effect of Si between both experiments could be explained by the fact that in the present study the low N fertilization (60 kg N ha^−1^) was applied over a long period, which has mainly limited the growth of plants cultivated under field conditions. Indeed, Good et al. [23] reported that beneficial micronutrients like Si provide no developmental advantage to plants grown under low availabilities of essential macronutrients such as N, P, and K.

This study observed that Si supply in plants cultivated with the highest dose of N fertilizer (160 kg N ha^−1^; 160 N + Si) increased the percentage of green leaves on plants until at least the flowering stage (84.85%) compared to control plants (75.73%), while the biomass of total leaves was not significantly different between either of the Si treatments (Figure 2). Thus, this result suggests that Si supply increases the leaf life span which in agreement with previous studies showing that Si supply delays leaf senescence in several plant species such as *Arabidopsis thaliana*, *Sorghum bicolor*, and *Brassica napus* [10,24]. Moreover, at the final stage of development, Si supply led to a significant increase in whole plant biomass (which might be due to the increase of the root and pods biomasses; Figure 3A) and an increase in the total N amount (+12.8%) in +Si supplied plants (Figure 3B; Table 2). This increase in the N amount without N concentrations changes (Figure 3C) was also highlighted thanks to the agronomic nitrogen recovery (ANR) index, which indicates the efficiency of plants in taking up N from the soil. Indeed, the ANR of 160 N + Si plants was significantly higher than in 160 N plants (57% compared to 43%). This increase in N uptake in 160 N + Si plants could be related to the increase in root biomass, which might improve the ability of Si-treated plants to prospect for N in the soil. As previously reported by several studies [10,25], it is also possible that Si promotes root expression of genes encoding nitrate transporters (*NRT* family), which would have allowed an improvement in N uptake, especially in plants cultivated with the higher N input (160 kg ha^−1^). Another assumption is that Si is able to increase N availability in the soil. This is reinforced by several works demonstrating that Si is able to modify physicochemical (such as soil exchange capacity) [26,27] and biological properties of soil (for example, by increasing the biomass of microbial nitrogen fixers) [28] and to improve uptake and accumulation of macronutrients and micronutrients in various plant species [29,30,31,32,33]. As an example, Singh et al. [34] reported that Si application in rice enhances the availability of N in soil and leads to an increase in uptake of this nutrient. From the evidence of these previous studies, the Si supplied here (12 kg ha^−1^) may have increased availability of N in the soil, improved N uptake, and led to an accumulation of N in the plants cultivated with the higher dose of N fertilizer (160 kg ha^−1^). Furthermore, our experiments showed that Si supply resulted in a significant increase in seed yield (4.2 T ha^−1^) of 16.6% under 160 N + Si compared to the 160 N plants (3.6 T ha^−1^). This result agrees with recent observations by Kuai et al. [35] in which two genotypes of *Brassica napus* cultivated under field conditions showed that Si supplied via spraying resulted in yield improvement. Interestingly, in our study, the increase in yield in 160 N + Si plants was associated with a preservation of seed yield quality components such as thousand-seed weight or the oil, glucosinolate, and protein concentrations, and the linoleic acid (ω*-*6): linolenic acid (ω*-*3) ratio was also not significantly different between seeds from plants cultivated with 160 kg N ha^−1^ and supplied or not with Si (Table 2). Moreover, there was no significant difference in the residual N in leaves of these plants suggesting that Si has no impact on N remobilization from leaves towards the seeds. This result is in agreement with the nitrogen harvest index (NHI), a component that indicates efficiency of N plant remobilization to the seeds, which was similar between the 160 N and 160 N + Si plants (Figure 5C). The increase in seed yield (with a preservation of N content in seeds) would therefore be due to a better usage of the N taken up by the 160 N + Si plants. This was confirmed by the agronomic efficiency (Figure 5A), an index that measures the efficiency of converting the N supply into seed yield, which was 1.5-fold higher for 160 N + Si than 160 N plants. This means that 1 kg of N supplied with Si enables the production of 14.2 kg of seeds against only 9.3 kg of seeds when N is supplied alone. This study indicated that the provision of Si was associated with better usage of N in rapeseed when it was provided with a higher dose of fertilizer and that it might be possible to reduce N inputs without affecting the yield of the crop. 

In addition, Si supply has a positive effect on micronutrient concentrations in seeds. Indeed, Si supply resulted in increases in nickel (Ni) concentration, but only in seeds from plants cultivated under the lower N fertilization regime (60 kg N ha^−1^; Figure 4D). Under both levels of N fertilization (60 or 160 kg N ha^−1^) there was an increase in iron (Fe) and cobalt (Co) concentrations in the seeds (Figure 4B,C). Although an improvement in remobilization/allocation of these nutrients due to additions of Si cannot be excluded, the increase in nutrient concentrations in seeds might be a consequence of stimulated root uptake as demonstrated for Fe and nitrate in *Brassica napus* and *Valerianella locusta* L. [10,25]. These results are particularly interesting because both Fe and Co are important for plant metabolism [36] but also for mammalian nutrition and health [37]. Indeed, Fe is involved in the synthesis of hemic enzymes such as hemoglobin and myoglobin [38,39] and Co is required for the B12 vitamin. Moreover according to the World Health Organization [40], Fe is one of the most deficient nutrients in the diet of more than half of the world’s population, and increasing it in plant-based foods (so-called biofortification) could overcome this deficiency, as previously suggested by Billard et al. [41]. 

In conclusion, this study has established that supplying Si under field conditions leads to better N efficiency in *Brassica napus* when the crop is cultivated with a 160 kg N ha^−1^ input enabling good growth and development. In our conditions of N inputs, this improvement in N efficiency is mainly due to better N uptake, but not better N remobilization, and increases yield while preserving seed quality. In addition, this study indicates the potential for combining N and Si fertilizers to promote mineral biofortification in *Brassica napus* seeds. 

## Figures and Tables

**Table 1:** Physical and chemical characteristics of the soil in the lysimeters. nd: non-detected value.

Depth (cm)	Particle Size Distribution (%)			pH (Water)	Total S (mg S g^−1^)	Organic C (mg C g^−1^)	Total N (mg N g^−1^)	Inorganic N (µg N g^−1^)	C:N Ratio	CEC (mol kg^−1^)	CaCO_3_ (%)
	Sand	Silt	Clay								
0–20	32	38	30	7.9	0.1	25.4	3.2	7.4	7.9	16.5	10
20–40	34	38	28	8.1	0.1	15.0	1.9	7.5	8.0	11.5	12
40–65	41	35	24	8.3	0.1	7.6	0.9	4.5	8.2	7.2	28
65–100	49	34	17	8.4	nd	3.4	0.4	4.7	8.5	4.6	38

**Table 2:** Yield component, concentration of oil, protein and glucosinolate concentrations and linoleic acid (ω-6): linolenic acid (ω-3) ratio (ω-6: ω-3 ratio) in mature seeds from rapeseed cultivated with 60 (60 N) or 160 kg of N ha^−1^ (160 N) and supplied with or without Si (12 kg ha^−1^: 60 N + Si and 160 N + Si). Values correspond to the mean ± SE for *n* = 3. The data obtained for seeds from plants treated with silicon (60 N + Si and 160 N + Si) were compared to their respective control * indicates significant difference at *p* ≤ 0.05.

	Yield (kg ha^−1^)	Thousand-Seed Weight (g)	Glucosinolate Concentration (µg g^−1^ DW)	Oil Concentration (% DW)	*ω-*6:*ω-*3 Ratio	Protein Concentration (% DW)
60 N	2696.96 ± 217.90	5.27 ± 0.33	13.10 ± 0.85	49.70 ± 0.96	2.37 ± 0.27	14.77 ± 0.13
60 N + Si	3122.13 ± 74.37	4.92 ± 0.21	15.43 ± 0.55	50.45 ± 0.55	2.42 ± 0.13	14.58 ± 0.45
160 N	3644.37 ± 182.48	4.81 ± 0.40	17.97 ± 0.66	47.4 ± 0.67	2.48 ± 0.06	16.50 ± 0.10
160 N + Si	4247.20 ± 75.23 *	4.54 ± 0.28	16.53 ± 1.54	47.93 ± 0.42	2.24 ± 0.10	17.33 ± 0.26

## Supplementary Materials

The following are available online at https://www.mdpi.com/2223-7747/8/5/137/s1, Figure S1: Mean monthly temperature and precipitation recorded at the meteorological station during the 2016–2017 growth season Arrows indicate main experimental steps, Figure S2: Total dry weight (A) and total nitrogen amount (B) of vegetative parts and seeds of rapeseed plants cultivated without N supply and harvested at T2 (G5 stage). Values correspond to the mean ± SE for *n* = 3, Table S1: Macro (grey) and micronutrient (white) concentrations in mature seeds from rapeseed plants harvested at T2 (G5 stage). Plants were cultivated in lysimeter with 60 (60 N) or 160 kg of N ha ^−1^ (160 N) and supplied with or without Si (12 kg of Si ha ^−1^; 60 N + Si 160 N + Si). Values represent the mean ± SE for *n* = 3.
