# Supplementary material for: Silicon Promotes Agronomic Performance in Brassica napus Cultivated under Field Conditions with Two Nitrogen Fertilizer Inputs
Source: Plants (Basel). 2019 May 22;8(5):137. doi: 10.3390/plants8050137 (PMC6571727; doi:10.3390/plants8050137)
Supplement: Supplementary file 1 [file plants-08-00137-s001.pdf]

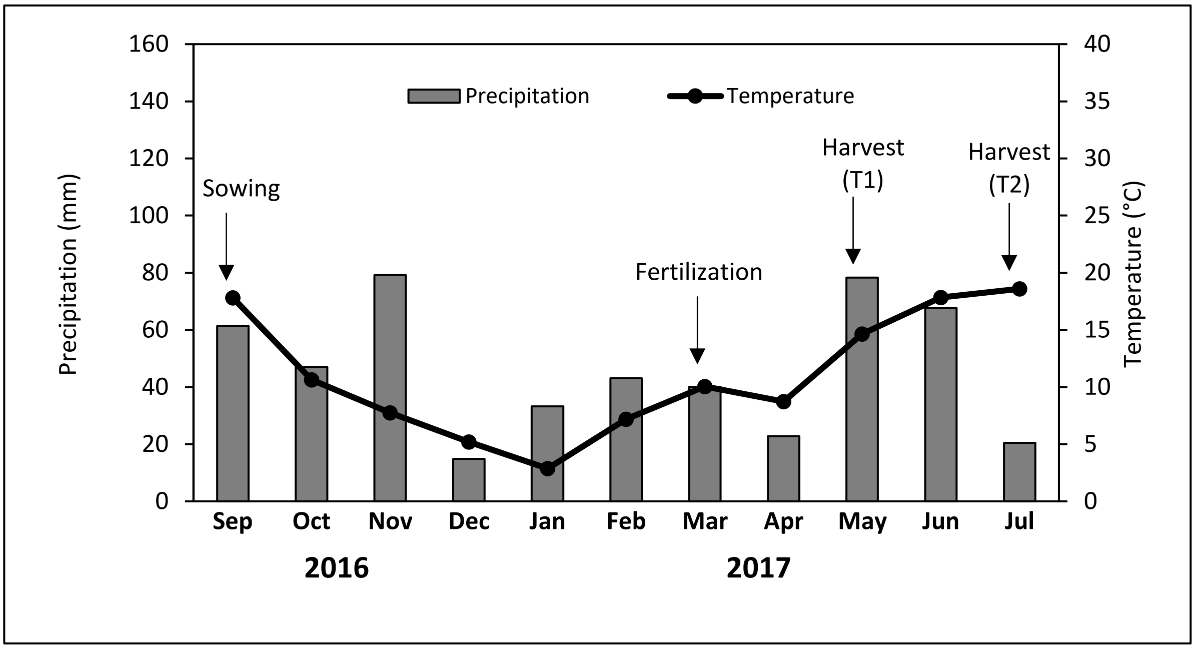

Figure S1. Mean monthly temperature and precipitation recorded at the meteorological station during the 2016–2017 growth season**.** Arrows indicate main experimental steps.

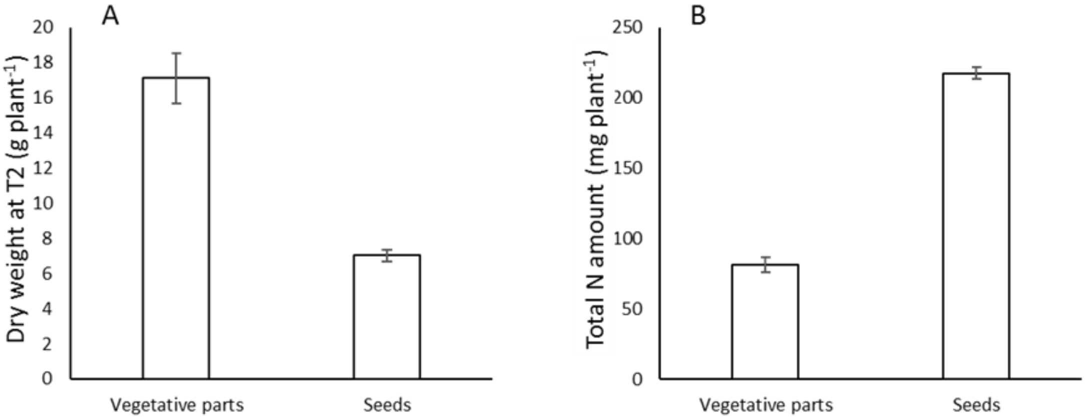

Figure S2. Total dry weight **(A)** and total nitrogen amount **(B)** of vegetative parts and seeds of rapeseed plants cultivated without N supply and harvested at T2 (G5 stage). Values correspond to the mean ± SE for $n = 3$.

Table S1. Macro **(grey)** and microelement **(white)** concentrations in mature seeds from rapeseed plants harvested at T2 (G5 stage). Plants were cultivated in lysimeter with 60 (60 N) or 160 kg of N ha^{-1} (160 N) and supplied with or without Si (12 Kg of Si ha^{-1}; 60 N + Si, 160 N + Si). Values represent the mean ± SE for *n* = 3.

Nutrient	60 N	60 N + Si	160 N	160 N + Si
Ca (mg g^{-1})	3.89 ± 0.08	3.87 ± 0.18	3.61 ± 0.19	3.52 ± 0.21
K (mg g^{-1})	5.61 ± 0.39	5.34 ± 0.20	5.72 ± 0.04	5.61 ± 0.15
Mg (mg g^{-1})	1.83 ± 0.07	1.74 ± 0.07	1.82 ± 0.06	1.72 ± 0.04
P (mg g^{-1})	4.12 ± 0.13	3.93 ± 0.20	3.62 ± 0.17	3.54 ± 0.09
S (mg g^{-1})	3.04 ± 0.05	2.86 ± 0.08	3.45 ± 0.12	3.17 ± 0.11
B (mg g^{-1})	11.22 ± 0.25	10.84 ± 0.14	11.73 ± 0.33	11.30 ± 0.55
Cu (mg g^{-1})	2.61 ± 0.07	2.58 ± 0.06	2.90 ± 0.35	2.79 ± 0.17
Mn (mg g^{-1})	25.46 ± 0.61	25.40 ± 1.16	27.26 ± 0.58	26.10 ± 0.61
Mo (mg g^{-1})	0.38 ± 0.06	0.40 ± 0.02	0.35 ± 0.01	0.35 ± 0.02
Se (mg g^{-1})	37.20 ± 0.66	30.21 ± 4.27	25.80 ± 3.37	29.90 ± 1.85
Zn (mg g^{-1})	28.77 ± 0.64	27.89 ± 0.66	36.43 ± 5.33	33.41 ± 4.13
